# Passive listening to preferred motor tempo modulates corticospinal excitability

**DOI:** 10.3389/fnhum.2014.00252

**Published:** 2014-04-24

**Authors:** Kelly Michaelis, Martin Wiener, James C. Thompson

**Affiliations:** Department of Psychology, George Mason UniversityFairfax, VA, USA

**Keywords:** transcranial magnetic stimulation, rhythm perception, tempo and timing, corticospinal excitability, individual differences

## Abstract

Rhythms are an essential characteristic of our lives, and auditory-motor coupling affects a variety of behaviors. Previous research has shown that the neural regions associated with motor system processing are coupled to perceptual rhythmic and melodic processing such that the perception of rhythmic stimuli can entrain motor system responses. However, the degree to which individual preference modulates the motor system is unknown. Recent work has shown that passively listening to metrically strong rhythms increases corticospinal excitability, as indicated by transcranial magnetic stimulation (TMS). Furthermore, this effect is modulated by high-groove music, or music that inspires movement, while neuroimaging evidence suggests that premotor activity increases with tempos occurring within a preferred tempo (PT) category. PT refers to the rate of a hypothetical endogenous oscillator that may be indicated by spontaneous motor tempo (SMT) and preferred perceptual tempo (PPT) measurements. The present study investigated whether listening to a rhythm at an individual’s PT preferentially modulates motor system excitability. SMT was obtained in human participants through a tapping task in which subjects were asked to tap a response key at their most comfortable rate. Subjects listened a 10-beat tone sequence at 11 log-spaced tempos and rated their preference for each (PPT). We found that SMT and PPT measurements were correlated, indicating that preferred and produced tempos occurred at a similar rate. Crucially, single-pulse TMS delivered to left M1 during PPT judgments revealed that corticospinal excitability, measured by motor-evoked potentials (MEPs), was modulated by tempos traveling closer to individual PT. However, the specific nature of this modulation differed across individuals, with some exhibiting an increase in excitability around PT and others exhibiting a decrease. These findings suggest that auditory-motor coupling induced by rhythms is preferentially modulated by rhythms occurring at a preferred rate, and that individual differences can alter the nature of this coupling.

## Introduction

Rhythm and time play an essential role in many of the behaviors we engage in every day. This is especially apparent in behaviors that require coordination between movements and auditory stimuli, such as playing an instrument, dancing, or simply talking in turn during conversation (McAuley et al., 2006; Zatorre et al., 2007). Evidence for a privileged link between auditory and motor systems exists in many forms: the ability to tap along with a beat appears to be universal among humans (Nettl, 2000); musical rhythms aid movement and coordination among Parkinson’s and stroke patients (McIntosh et al., 1997; Thaut et al., 1997; Rodriguez-Fornells et al., 2012; Nombela et al., 2013), and the emotional salience or “groove” rating of a musical excerpt can influence motor system excitability (Kornysheva et al., 2010; Giovanelli et al., 2013; Stupacher et al., 2013). However, the degree to which individual preferences influence audiomotor linkages remains relatively unexplored. Recent results from both functional Magnetic Resonance Imaging (fMRI) and transcranial magnetic stimulation (TMS) studies identify metrical saliency, or the strength of the underlying beat, as driving increases in neural activation and excitability in motor areas (Zatorre et al., 2007; Cameron et al., 2012), and additional fMRI evidence demonstrates that increases in ventral premotor cortex activation are associated with individual preference for slow vs. fast tempos (Kornysheva et al., 2010). In order to further our understanding of how the motor system responds to individual differences in the perception and preference of rhythmic auditory stimuli, the present study examined changes in motor system excitability while subjects listened to an individually preferred tempo (PT).

Specific properties of auditory stimuli have been shown to modulate neural response (Zatorre et al., 2007; Chen et al., 2008a; Bengtsson et al., 2009; Kornysheva et al., 2010; Giovanelli et al., 2013); for example, Kornysheva et al. (2010) demonstrated that tempo is one such property. The authors conducted an fMRI experiment in which subjects were presented with a range of auditory sequences that consisted of five properties (tempo, measure, beat subdivision, rhythmic figure, and timbre) that were orthogonally varied on two or three levels (i.e., fast vs. slow tempo). In a forced-choice paradigm, subjects were asked to make either an aesthetic judgment (“beautiful?”) or a tempo judgment (“fast?”); before fMRI analysis, a linear regression model was used to group subjects according to the most significant predictors of variance (tempo and timbre) such that each subject could be classified as having a tempo preference (“fast” vs. “slow”) and a timbre preference (“rock” vs. “bongo”). During the aesthetic judgment task, BOLD activity in left ventral premotor cortex (PMv) increased when subjects heard sequences at a PT. In addition, the magnitude of the activity increase was correlated with the strength of the subjects’ preference for that tempo category. A follow-up TMS study using the same rhythmic stimuli expanded these results by showing that not only is PMv activity correlated with hearing a PT category, but inhibitory repetitive TMS over PMv temporarily reduces tempo preference strength and stability as measured through behavioral ratings (Kornysheva et al., 2011). These findings demonstrate that hearing a PT category preferentially influences motor system activity and that PMv is involved in assigning this tempo preference.

What drives this effect of tempo preference? In an effort to further understand how the motor system may be influenced by auditory tempos, the present study examined whether an individually preferred beat rate could preferentially modulate motor response when compared to similar beat rates. As discussed by Large and Jones (1999) and McAuley et al. (2006), an individually preferred beat rate, or PT, arises from dynamic attending theory and refers to the rate of a putative endogenous oscillator. In order to attend to an event, attention must be allocated both to the correct space and to the correct instant in time (McAuley et al., 2006). Large and Jones (1999) describe how the ability to attend to external events in time may arise from internal “attending rhythms” that are “capable of entraining to external events and targeting attentional energy to expected points in time”. If attention flows from an endogenous rhythmic oscillator in this manner, then predictable periodic events should be processed more efficiently because attention can be more easily applied to them. A recent event-related potential (ERP) study supports this assertion and demonstrates that dynamic attending theory has implications for auditory-motor coupling: ERP results showed that encoding of auditory sequences was stronger when stimuli were synchronized with the subject’s motor movements (Schmidt-Kassow et al., 2013). McAuley et al. (2006) also discuss the concept of rhythmic attending and its reliance on an internal oscillator. Here, the oscillator rate is thought to contribute to an individualized preference for beat rate. In this paradigm, the rate of the intrinsic oscillator, or an individual’s PT can theoretically be indexed through performance on both motor and perceptual tasks. Because this oscillator is thought to be a central mechanism, the tempo produced during an unguided isochronous tapping task, referred to as spontaneous motor tempo (SMT), should be highly correlated with PT in a passive listening task, referred to as preferred perceptual tempo (PPT). Behavioral measures collected from 305 participants ranging in age from 4 to 95 years demonstrated that SMT and PPT were indeed strongly correlated, and furthermore, individual PT slows throughout the lifespan (McAuley et al., 2006), suggesting that individual differences in PT may contribute to tempo preference effects on motor system excitability.

The investigation of PT on motor system response is best understood within the context of auditory-motor coupling, or the idea that the auditory and motor systems are preferentially linked, such that auditory stimuli can prime the motor system to respond. As mentioned above, fMRI investigations have provided correlational evidence of this audio-motor link. In a study by Chen et al. (2006), subjects were asked to tap along with six different isochronous tone sequences, each with a varying degree of metrical saliency. Results showed that as metrical strength increased, subjects showed greater blood-oxygenation-level dependent (BOLD) activation in superior temporal gyrus and dorsal premotor cortex, as well as an increase in the functional connectivity between these two regions. Similarly, a previous TMS study investigated the modulatory effect of single-pulse TMS delivered on and off the beat during both metrically strong and metrically weak tone sequences (Cameron et al., 2012). The high temporal resolution of online TMS pulses presents a useful method for measuring motor system excitability at specific points in time. Pairing TMS pulses with electromyography (EMG) readings allows for the measurement of motor-evoked potentials (MEPs), which have been shown to reflect activity in other motor regions like premotor and supplementary motor areas (Hanakawa et al., 2009). Using this paradigm, Cameron et al. (2012) found greater corticospinal excitability (higher peak-to-peak MEP amplitude) for TMS pulses delivered on the beat of metrically strong sequences, but not for metrically weak sequences or for pulses delivered off the beat. Although this study had a very small sample size (*n* = 4), it is worth noting that they also reported individual differences in corticospinal excitability in response to metrical stimuli. While three of the four participants exhibited greater excitability for beat-synchronized TMS pulses delivered during metrically strong as opposed to metrically weak tone sequences, one participant showed the opposite, exhibiting greater excitability when TMS was applied on the beat of metrically weak sequences. The authors acknowledge the difficulty of interpreting these differences due to the small sample size, but they suggest individual variations in attention or “effortful listening” may have been a contributing factor. A similar TMS paradigm employed by Stupacher et al. (2013) also reported individual differences in motor cortex response to rhythmic stimuli. Specifically, they assessed how the “groove” of a musical clip, or how much the music inspires movement (e.g., foot tapping), may influence motor system excitability. Musicians and non-musicians listened to low-groove and high-groove music clips while receiving single-pulse TMS and EMG recordings were taken from the right hand and forearm. Compared to MEPs for spectrally matched noise, musicians exhibited greater peak-to-peak MEP amplitudes for high-groove music when TMS was delivered on the beat vs. off the beat; however, for non-musicians, MEP amplitudes were reduced for high-groove music regardless of whether or not the pulses were synchronized with the beat. Additionally, compared to musicians, non-musicians exhibited higher pre-pulse EMG activity when listening to high-groove music. These differences indicate that while groove does affect motor system response, the nature of this response may differ between individuals. What remains unresolved is the degree to which individual preference modulates excitability in the motor system.

Drawing on the body of literature surrounding auditory-motor coupling and PT, the present study examined whether an individual’s preferred beat rate preferentially modulates corticomotor excitability over other beat rates. Using the same measures of tempo preference as McAuley et al. (2006), we assessed SMT for each subject through an isochronous tapping task. Subjects then listened to 10-beat tone sequences traveling at a range of tempos relative to their SMT (5 faster than SMT, 5 slower than SMT, and 1 equal to SMT). While listening to these 11 tempos, subjects were asked to rate each one according to how “right” it felt to them, and these ratings were used to generate each subject’s PPT. Given the high temporal resolution provided by TMS, the current study employed an online TMS paradigm similar to Stupacher et al. (2013). While listening to each tempo, beat-synchronized single-pulse TMS was delivered over left M1 and MEPs were recorded from the first dorsal interosseous (FDI) muscle of the right hand. Based on the findings discussed above, we expect to see an effect of PT on changes in MEP amplitude.

## Methods

### Participants

Fourteen adults (7 female), between 18 and 35 years of age were included in the study. All participants were right-handed as measured by the Edinburgh Handedness Inventory (Oldfield, 1971), and each gave informed consent according to a University-approved protocol. 8 participants (3 female) reported varying degrees of musical experience ranging from 1 to 15 years (*M* = 6.2 years, *SD* = 4.5). The age at which they began playing ranged from 8 to 13 (*M* = 10.3, *SD* = 1.9), and examples of instruments played include piano, guitar, and tuba. Of these eight, only three were currently playing at the time of the study. The remaining six participants reported no musical experience. The experiment was approved by the local ethics committee and performed according published TMS safety regulations (Rossi et al., 2009).

### SMT

Immediately prior to the TMS session, a measure of each subject’s SMT was obtained through a self-paced tapping task. Subjects were seated in front of a computer and asked to tap a response key at whichever rate felt “most comfortable”. Subjects were instructed to keep a steady pace and to make the spaces between taps as even as possible. Each subject completed 124 taps, broken up into 4 blocks of 31 taps each (30 time intervals). The first tap of each series was removed, and individual SMT was calculated as the average inter-tap interval across the 4 blocks (McAuley et al., 2006; Wiener et al., 2011).

### Auditory Stimuli and PPT

Auditory stimuli consisted of an isochronous 10-beat tone sequence delivered at 11 different tempos. The timbre and pitch of the tone was designed to match the sound created by the TMS pulse and was kept constant. We modified the process used by McAuley et al. (2006) and determined the rates of the 11 tempos using the subjects’ individual SMTs: 5 tempos traveled at a rate faster than SMT, 5 traveled slower than SMT, and 1 traveled at the same rate as SMT. The faster and slower tempos were logarithmically spaced around SMT so that each subject was presented with a sufficient range of tempo stimuli relative to his or her own SMT. The five slower tempos were presented such that tempo (T) was equal to values of T/SMT of 1.221, 1.49, 1.82, 2.224, and 2.718; the five faster tempos had T/SMT values of 0.818, 0.68, 0.549, 0.449, and 0.367. Following each auditory sequence, subjects were presented with a 21-point rating scale (min = −10, max = 10) and were asked to rate each sequence according to how comfortable the tempo felt to them. Tempos that felt “too slow” were to be assigned a value between −10 and −1, tempos that felt “too fast” a value between 1 and 10, and tempos that felt “just right” a value of 0. Each of the 11 tempos was presented 10 times in random order, for a total of 110 trials with 10 beats each. Stimulus timing and presentation were controlled via a laptop computer connected to an external monitor using Python extensions provided by Psychopy, version 1.75 (Peirce, 2009).

### TMS and EMG recordings

**Figure 1:**
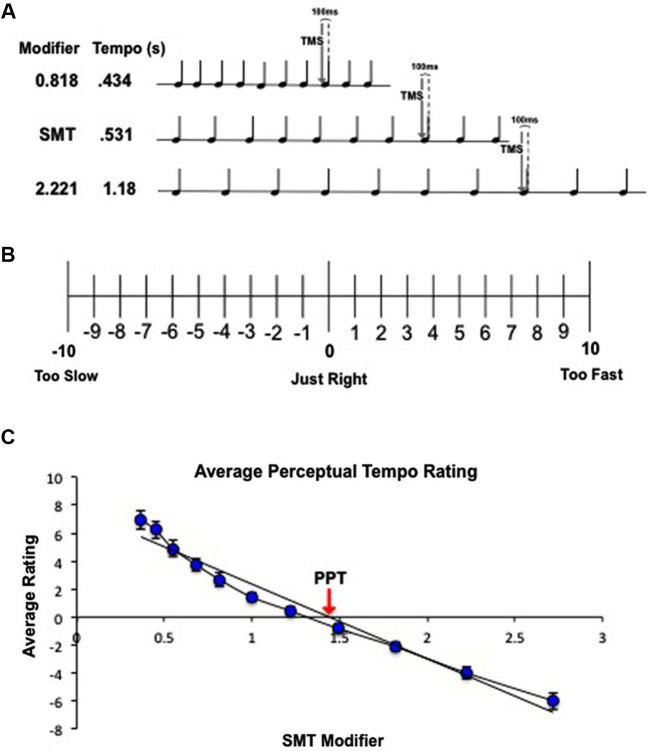
**(A)** Schematic of combined behavioral/TMS task for a representative subject with an SMT of 0.531 s. Subjects passively listened to 10-beat tone sequences traveling at a range of tempos relative to their SMT. Tempos were created by multiplying the subject’s SMT by 10 logarithmic modifiers to create 5 faster tempos and 5 slower tempos. While subjects listened to each tone sequence, single-pulse TMS was delivered over left M1 100 ms before the onset of the eighth beat. **(B)** Behavioral ratings to determine PPT. Subjects listened to tone sequences traveling at 11 different tempos, one at the subject’s SMT and 10 tempos spaced logarithmically above and below SMT (1 = SMT). At the end of each tone sequence, subjects rated the tempo according to how “right” the speed felt to them using the scale pictured above. **(C)** Calculation of PPT for a single subject. Ratings were averaged across trials and fit with a linear regression. The zero crossing indicates the subject’s PPT.

Single-pulse TMS was delivered using a Magstim Rapid 2 stimulator (Magstim, Whitland, UK) connected to a 70 mm figure of eight coil. High resolution, T1-weighted structural MRI scans were acquired prior to participation, and neuronavigation was achieved using Brainsight (Rogue Resolution, Montreal, Canada) targeting software. TMS was delivered over left M1. The exact location within M1 was determined as the point at which a TMS pulse reliably produced an MEP from the right hand. Resting motor threshold (RMT) was determined as the stimulation level at which 50% of tested pulses elicited a hand movement. Once determined, stimulation level was set to 110% RMT. Similar to previous work combining TMS and EMG, EMG recordings were taken from the FDI muscle of the right hand and changes in MEPs were used as an index of corticospinal excitability (Giovanelli et al., 2013; Stupacher et al., 2013). The EMG signal was recorded using disposable Ag/AgCl electrodes placed on the forefinger and purlicue, with a ground electrode placed on the back of the hand. EMG recordings were collected using a Biopac MP150 amplifier (Biopac, Goleta, CA) with a sample rate of 20,000 Hz onto a separate computer running AcqKnowledge software, version 3.9.1; TMS pulses elicited digital triggers to the Biopac amplifier via a Bayonet Neill-Concelman cable connection. Subjects sat upright in a cushioned chair with armrests and a headrest and were instructed to keep the right arm relaxed on the armrest with the palm up. Subjects listened to the auditory stimuli through earplug headphones (Plugfones) at a comfortable volume. TMS was delivered 100 ms prior to the onset of the eighth beat of each 10-beat tone sequence (110 pulses total). Motor cortex excitability has been shown to be maximal just before a predicted beat (Cameron et al., 2012), thus the 100 ms offset was included so that the TMS pulse coincided with peak motor cortex excitability. After passively listening to each tone sequence, subjects clicked an onscreen ratings scale by moving a mouse with the left hand (keeping the right hand relaxed). A schematic of the combined behavioral/TMS task can be seen in Figure 1. The experiment lasted about 1 h from beginning to end.

## Data Analysis

### PPT Calculation

PPT was calculated for each subject. Following the analysis procedure used by McAuley et al. (2006), ratings for each of the 11 tempos were averaged across trials and individually fit with a linear regression for each subject (Figure 1C). The zero crossing of the regression line was taken as the predicted modifier value for the tempo that received the most zero ratings (a “just right” tempo). This modifier value was multiplied by the subject’s SMT to convert it into seconds, and the result was taken as the subject’s PPT.

### MEP Calculation

EMG recordings were epoched offline using Matlab (Mathworks) software. MEPs were calculated by baseline-correcting post-TMS EMG data to the time period 50 ms prior to the TMS pulse. MEPs were calculated as the peak-to-peak amplitude difference between 10 and 80 ms following TMS, in accordance with standard methodology (Rösler and Magistris, 2008). Since substantial inter-individual variability exists in the size of MEPs (Wasserman, 2008), we normalized all MEP measures for each subject by their average peak-to-peak amplitude, reducing each set to a mean of one. As such, MEPs above one represent relatively greater corticospinal excitability, whereas values below one indicate a relative suppression of activity. MEPs exceeding 2 SDs from the mean were removed for each subject.

### Quadratic Fitting and Segregation

Due to our a-priori hypotheses regarding the effect of tempo differences on MEP size, for each subject, normalized average MEP amplitudes at each tempo were fit with a quadratic curve (MEPa*t2 + MEPb*t + MEPc), where *t* represents the tempo modifier and *MEPa*, *MEPb*, *MEPc* and represent the first, second and third constants. Individual subject curves were then segregated according to the sign of the first constant, which indicated whether the shape of the curve was concave (MEPs are reduced closer to SMT) or convex (MEPs are increased closer to SMT) and averaged within groups.

## Results

### SMT and PPT

**Figure 2:**
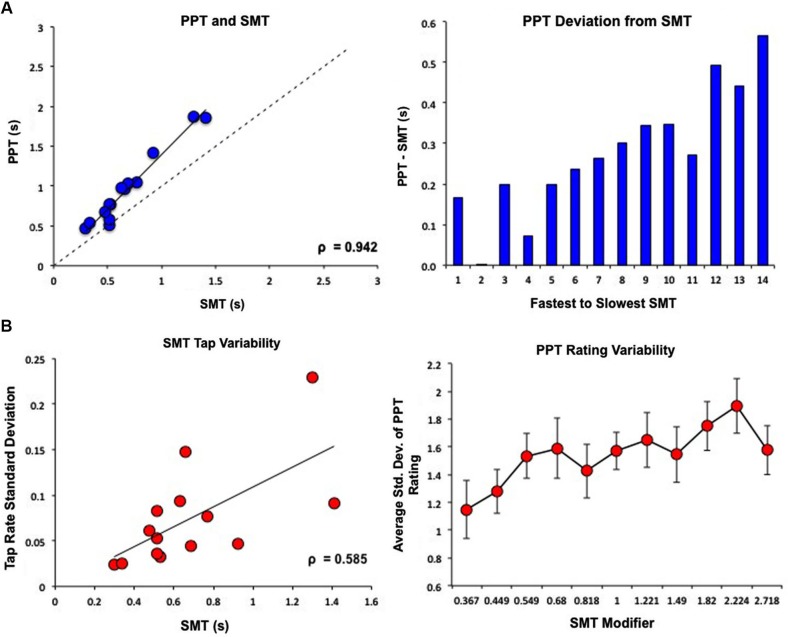
**Behavioral results of PPT and SMT for all subjects**. SMT and PPT values are displayed as seconds between tones/taps. **(A)** PPT and SMT were highly correlated. However, subjects consistently rated PPT as slower than SMT, with larger deviations between PPT and SMT for subjects with slower SMTs. **(B)** Variability in behavioral results of PPT and SMT. On the *x*-axis of the graph on the right, “SMT Modifier” refers to the values that each subject’s SMT was multiplied by to create the log-spaced tempos of the tone sequences. This means that 1 = SMT, smaller values designate faster tempos, and higher values designate slower tempos. These modifier values were the same for each subject while the actual tempos changed depending on the subjects’ SMTs. Subjects with slower SMTs showed greater variability in tap rate. Similarly, subjects were more variable in their perceptual ratings of slower tempos.

Subject’s SMT values, or average inter-tap intervals, ranged from 0.298–1.41 s (*M* = 0.68 s, *SD* = 0.32). One subject exhibited abnormally high tapping and rating variability, exceeding 2 SDs from the group average, and was removed as an outlier. Subject’s PPT values ranged from 0.463–1.87 s (*M* = 0.98 s, *SD* = 0.45). SMT and PPT were highly correlated (Pearson’s *r* = 0.978, Spearman’s *ρ* = 0.942, *p* < 0.05), however, subjects consistently choose a PPT value that was slower than their SMT value, with larger deviations between PPT and SMT occurring at slower tempos (Figure 2A). Subjects with slower SMT values exhibited greater variability in tap rate (Pearson’s *r* = 0.64, Spearman’s *ρ* = 0.585, *p* < 0.05); notably, we found no correlation between the coefficient of variation for SMT, calculated by dividing the standard deviation in inter-tap interval by the mean inter-tap interval, and the SMT value (Pearson’s *r* = 0.064). Similarly, subjects were more variable in their perceptual ratings of slower tempos (*F*_(1,13)_ = 5.393, *p* < 0.05; Figure 2B).

### EMG Results

**Figure 3:**
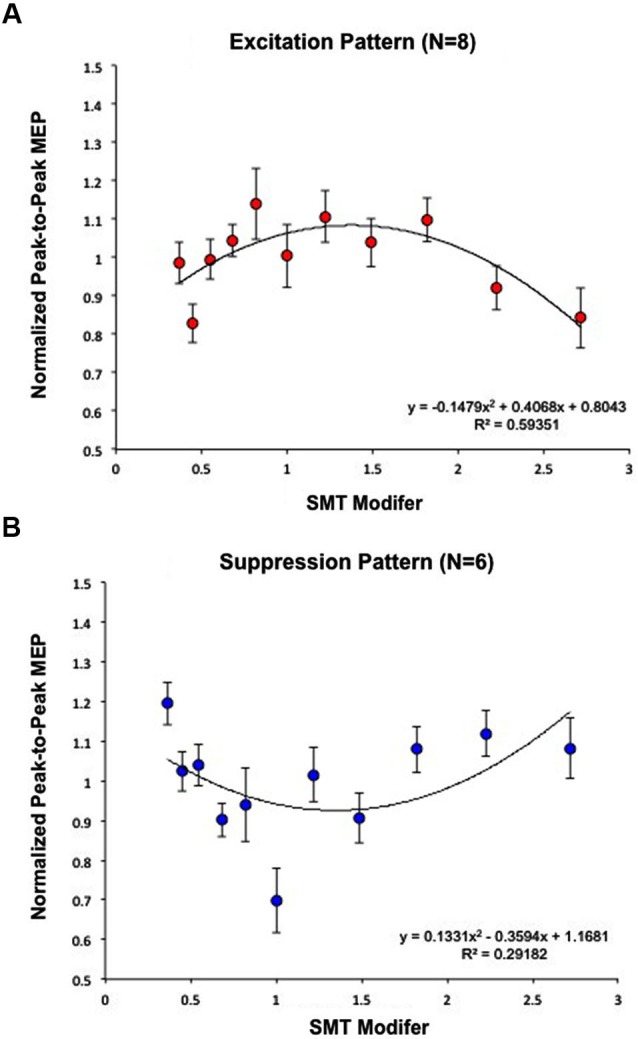
**Calculation of effects of PT on MEP modulation**. Subjects were analyzed based on the fit of a quadratic model to EMG data and segregated according to the direction of the maximal change in MEP amplitude. **(A)** Eight subjects were best fit by a negative curve (Excitation Pattern), meaning MEP amplitudes were maximal closest to SMT (1 = SMT). **(B)** Six additional subjects exhibited a positive curve (Suppression Pattern), meaning MEP amplitudes were minimal closest to SMT. Together, these graphs demonstrate that listening to PT modulates MEP amplitude, but that the direction of change differs across individuals. As in **Figure 2B**, “SMT modifier” refers to the values that each subjects’ SMT was multiplied by to create the log-spaced tempos of the tone sequences. This means that 1 = SMT, smaller values designate faster tempos, and higher values designate slower tempos. These modifier values were the same for each subject while the actual tempos changed depending on the subjects’ SMTs.

Each subject’s normalized average MEPs were fit by a quadratic curve. Eight subjects were well fit by a negative (concave) curve (Excitation Pattern) [Within-subject contrast, *F*_(1,7)_ = 33.680, *p* < 0.05] (*R*^2^ = 0.59); indicating that MEP amplitudes were maximal closest to SMT (Figure 3A). Six additional subjects exhibited a positive (convex) curve (Suppression Pattern) [*F*_(1,5)_ = 9.106, *p* < 0.05] (*R*^2^ = 0.29); indicating that MEP amplitudes were minimal closest to SMT (Figure 3B).

**Figure 4:**
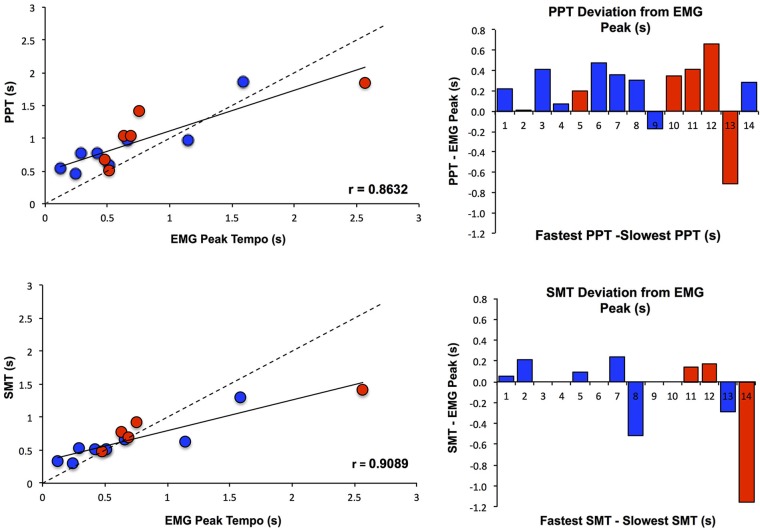
**Relationship between PT and MEP amplitude modulation**. PPT and SMT were each highly correlated with the tempo at which changes in MEP amplitude were maximal (EMG Peak), and subjects with slower SMT and PPT values exhibited greater deviations from EMG Peak. Subjects are color coded according to whether they showed an increase in MEPs when listening to PT (Excitation Pattern, shown in red) or a decrease in MEPs when listening to PT (Suppression Pattern, shown in blue). SMT, PPT and EMG Peak values are displayed as seconds between tones/taps.

To further assess the effect of PT on MEP amplitude, Pearson and Spearman correlations were calculated between the EMG peak tempo and both measures of PT (SMT and PPT). EMG peak tempo was defined as the tempo at which changes in MEP amplitude were maximal, regardless of the direction (excitation or inhibition). As Figure 4 shows, EMG peak tempo was strongly correlated with both SMT (Pearson’s *r* = 0.890, Spearman’s *ρ* = 0.906) and PPT (Pearson’s *r* = 0.849, Spearman’s *ρ* = 0.824), with larger deviations occurring at slower tempos. We also calculated the coefficient of variation in EMG amplitude at each tempo (standard deviation in MEP amplitude divided by average MEP amplitude); notably we found no relationship between SMT, PPT, or EMG peak tempos and the EMG coefficient of variation (Klein-Flügge et al., 2013).

### Non-parametric tests

Although both SMT and PPT exhibited strong correlations with EMG peak tempo across subjects, there remains the possibility that these correlations arose by chance. Specifically, EMG peak tempos were calculated by multiplying the modifier value where the peak MEP change occurred by an individual’s SMT. As such, any correlation between SMT and EMG peak tempos will already exhibit a higher correlation by chance. In order to test whether the correlations exhibited did not occur randomly, it was necessary to re-assess the level of chance through non-parametric means. Using Monte Carlo simulations, we generated 10,000 random EMG data sets, for the same number of subjects, based on the statistics of our original EMG data set (mean, SD), drawn from a uniform distribution, and for the same number of subjects. For each generated data set, we analyzed the data in the exact same manner as the original data set, by fitting each “subject” with a quadratic curve and using the sign of the first constant to determine whether to take the maximum or minimum value as the EMG modifier, then multiplying that modifier by the corresponding original SMT value, then correlating the resulting EMG peak tempos with SMT and PPT values. The 10,000 resulting Spearman *ρ*-values then served as the null distribution for correlations that resulted by chance for SMT and PPT. We chose a critical *α* of 0.05, one-tailed for testing significance. The result these simulations demonstrated that the observed Spearman *ρ*-value for both correlations exceeded the critical *α*-level for both SMT (critical *α* = 0.85) and PPT (critical *α* = 0.44) correlations, indicating that these correlations did not occur by chance.

## Discussion

It has been established that passively listening to a rhythmic auditory stimulus can affect motor cortex response, and that this response is sensitive to changes in stimulus properties like metrical strength, groove rating, and tempo category (slow vs. fast) (Zatorre et al., 2007; Bengtsson et al., 2009; Kornysheva et al., 2010; Cameron et al., 2012; Stupacher et al., 2013). The present study demonstrates that corticomotor excitability is also preferentially modulated by rhythms traveling at an individually preferred rate, consistent with the idea of an endogenous oscillator (McAuley et al., 2006). However, the nature of this modulation differs across individuals, taking the form of either increased or decreased excitability as the stimulus tempo approaches a subject’s PT.

In order to determine PT, the present study replicated the self-paced tapping and perceptual rating tasks employed by McAuley et al. (2006). Motor and perceptual measures of PT, SMT and PPT respectively, are thought to arise from the same hypothetical endogenous oscillator, and as such, they should be closely related. As expected, measures of SMT and PPT were highly correlated; however, for all subjects, PPT values were slower than SMT values, with the deviation between PPT and SMT increasing at slower tempos. These results are similar to those found by McAuley et al. (2006), which also showed a strong correlation and a trend toward slower PPT values, suggesting that with slower tempos there is greater difficulty in perceptually identifying tempo preference. We additionally observed that variability of both tap rates and perceptual ratings increased at slower tempos, aligning with scalar effects in timing (Gibbon et al., 1997), demonstrating a constant coefficient of variation across intervals. However, it is noteworthy that perceptual ratings were more variable with slower tempos in this regard, as these rating judgments simply measured the individual preference for a given tempo. Increasing variability for the PPT measurements with slower tempos then may also reflect greater uncertainty in the judgment of tempos traveling at a slower rate.

EMG results demonstrated that for all subjects, corticomotor excitability was modulated by passively listening to one’s own PT. This result is consistent with previous work demonstrating that listening to rhythmic stimuli is associated with increases in BOLD activation of PMv and SMA (Zatorre et al., 2007; Bengtsson et al., 2009), and further complements the results of Kornysheva et al. (2010), which showed that increases in PMv activation are modulated by listening to a PT category. In their follow-up TMS study, Kornysheva et al. (2011) demonstrated that stimulation of PMv alters subject’s behavioral preferences for slow and fast tempo categories; the present study compliments these results by demonstrating changes in M1 excitability in response to individually PTs. Additionally, EMG results from the present study revealed that there are individual differences in the nature of this modulatory effect of PT, with 8 of 14 subjects exhibiting an increase in excitability and six exhibiting a decrease in excitability when listening to their PT.

Why might subjects exhibit different patterns of excitation or suppression around PT? Previous work has shown that mental simulation of motor movements, or motor imagery, has an effect on motor cortex activation and excitability, and so motor imagery may have contributed to the individual differences described above. Fadiga et al. (1999) applied single-pulse TMS to motor cortex while subjects imagined opening and closing the hand and found that mental simulation of hand movements increased corticospinal excitability. Similarly, corticospinal excitability can be modulated by attention. Using a paired associative stimulation paradigm (PAS), Stefan et al. (2004) demonstrated that the degree of attention to a target hand modulated corticospinal response. PAS involves the combination of low frequency median nerve stimulation and single-pulse TMS in such a way that MEPs from the target muscle are increased. MEP amplitudes as evoked by PAS were measured relative to baseline in three attention conditions: (1) attention to the hand with vision of the hand occluded; (2) attention to an arithmetic task; and (3) attention to the target hand while also viewing the hand. Results showed that MEP amplitudes were modulated with the grade of attention to the hand, with decreased amplitudes when attention was diverted away, increased amplitudes when attention but not vision was on the hand, and even greater amplitudes when both attention and vision were on the hand.

In the present study, subjects were instructed to sit with their hands relaxed and listen to the auditory tone sequences. There were no specific instructions regarding attending to or away from the stimulated hand, but individual differences in attention to the tones, the surrounding room, or the stimulated hand likely affected the MEP amplitudes measured from each subject. Thus, differential levels of motor imagery and/or attention to the hand may have contributed to whether a particular subject showed an increase or decrease in excitability around PT. In this regard, it is noteworthy that our subjects responded with the opposite (non-stimulated) hand. One possibility, then, is that subjects who showed an increase in motor cortex excitability were implicitly simulating the action of tapping in the dominant hand; when the perceived beat matched the PT, the excitability of the hand area thus increased, in a manner consistent with embodied cognitive theories of action (Barsalou, 2008). The difference in excitability patterns between subjects also mirrors previous work demonstrating that participants who hear an implied beat during perceptual judgments also show increased premotor activation (Grahn and McAuley, 2009). Further investigation is necessary to assess the degree of this contribution, and future work will replicate the methods above while adding specific instructions regarding application of attention and motor imagery. Another perceptual phenomenon that may have influenced the outcome of the present study is subjective accenting, which refers to the unequal perception of equal tones in an isochronous sequence. As Brochard and colleagues demonstrate in their 2003 ERP study, the perception of alternatively strong and weak accented beats is related to dynamic oscillations in attention, resulting in a binary metrical structure in which odd-numbered tones are perceived as “accented” and even-numbered events are perceived as “unaccented” (Large and Jones, 1999; Brochard et al., 2003). In the present study, subjects received single-pulse TMS on the eighth beat of every 10-beat tone sequence. According to Brochard and company, the eighth beat corresponds to a “weak” or unaccented beat, and therefore to a trough rather than to a peak of attention. While it is uncertain how stimulating on an accented beat (i.e., the ninth beat) may have altered the present results, the fact that both subjective accenting and PT are thought to rely on the same dynamic fluctuations in attention suggests that the placement of TMS pulses within a sequence is an important consideration and should be incorporated into future studies.

Musical experience is another factor that may contribute to individual differences in the effect of PT. The impact of musical training on the auditory and motor systems has been well documented; musicians show a variety of structural differences, including increased cortical representation of motor areas specific for the hands and enhanced organization of auditory-motor connections, greater perceptual sensitivity to changes in auditory rhythms, and increased functional efficiency of motor networks (Jäncke et al., 2000; Gaser and Schlaug, 2003; Bengtsson et al., 2005; van Zuijen et al., 2005; Hyde et al., 2009; Herholz and Zatorre, 2012). Furthermore, the TMS investigation by Stupacher et al. (2013) found that the nature of changes in corticospinal excitability when listening to high groove music was dependent on musical experience: musicians exhibited and increase in excitability when listening to high groove music, while non-musicians exhibited a decrease. In addition to the musical training effects described above, the authors attribute these differences to a more refined motor action threshold in musicians. They posit that the greater refinement of motor networks and the common practice of movement simulation found among musicians leads to greater motor control with less effort; thus, musicians are better able to listen to a movement-inducing stimulus and remain still, without affecting corticospinal excitability. Without this refinement of the motor action threshold, non-musicians’ efforts to remain still when listening to a movement-inducing stimulus result in a suppression of corticospinal excitability (Stupacher et al., 2013). At the time of testing, only three subjects in the current study were actively playing instruments; of those three, two exhibited an excitation pattern around PT and one exhibited a suppression pattern. The other five participants with musical experience were also distributed across the excitation and suppression groups. Thus, while several of the subjects in the present study had differing levels of musical experience, it was not a controlled variable and there were no demonstrable effects of musical experience with respect to modulation direction (excitation vs. suppression of excitability). However, there is some indication that as little as 15 months of training may be sufficient to induce experience-dependent plastic effects, and that in some cases, simply listening to music can alter neural responses to auditory stimuli (Hyde et al., 2009; Herholz and Zatorre, 2012). Future studies of motor excitability should take these findings into consideration when selecting participants. Additionally, previous work has shown that individual differences exist in tempo preference strength (Kornysheva et al., 2010, 2011) and the ability to perceive a beat (Grahn and McAuley, 2009), and that these differences are correlated with changes in motor and premotor cortex responses. Therefore, future studies will implement measures of individual rhythmic ability and tempo preference strength (the extent to which an individual prefers his or her PT).

In conclusion, the present study demonstrates that corticomotor excitability is preferentially modulated by listening to an individually PT. Furthermore, there are individual differences in the nature of this effect: some people exhibit an increase in excitability as tempos near their PT, while others exhibit a decrease in excitability. These differences may be driven by factors like motor imagery, attention, musical experience, or rhythmic ability, though further investigation is necessary to determine the extent to which each factor contributes. By furthering our understanding of how the motor system responds to specific auditory stimuli, the present study represents an important addition to the body of literature surrounding auditory-motor coupling and temporal processing. Previous work has shown that the rhythmic complexity, metrical strength, emotional valence, tempo category, and groove of a stimulus can influence motor system response (Chen et al., 2008b; Kornysheva et al., 2010; Cameron et al., 2012; Giovanelli et al., 2013; Stupacher et al., 2013). The current results demonstrate a higher level of specificity with regard to tempo modulation of motor system response; namely, that an individually PT, which stems from the rate of a hypothetical endogenous oscillator, is capable of preferentially modulating motor system excitability when compared to similar tempos of similar speeds (McAuley et al., 2006). In clinical settings, the principles of auditory-motor coupling have informed music and rhythm-based therapies for stroke, traumatic brain injury, and Parkinson’s patients (McIntosh et al., 1997; Thaut et al., 1997; Rodriguez-Fornells et al., 2012; Nombela et al., 2013). In addition to their usefulness in research settings, the present findings regarding PT might improve the efficacy of these treatments.

## Conflict of interest statement

The authors declare that the research was conducted in the absence of any commercial or financial relationships that could be construed as a potential conflict of interest.
